# Supplementary material for: Juggling between caregiving and self-actualization: Older parents’ lifelong experience of caring for an adult child with developmental disabilities
Source: PLoS One. 2022 Nov 3;17(11):e0276779. doi: 10.1371/journal.pone.0276779 (PMC9632868; doi:10.1371/journal.pone.0276779)
Supplement: Attachment — Submitted filename: Theory hierarchy.docx [file pone.0276779.s001.docx]

Crotty, 1998 (*The foundations of social research: Meaning and perspective in the research process)*:

1. Epistemologies:
   - objectivism
   - constructivism
   - constructionism
   - subjectivism
2. Theoretical perspectives:
   - Positivism*,* Post-positivism
   - Interpretivism *(*Symbolic interactionism*;* Phenomenology*;* Hermeneutics)
   - Critical inquiry*,* feminism*,* postmodernism*,* etc.
3. Methodologies:

- Ethnography*,* phenomenological*,* grounded theory*,* heuristic inquiry*,* participatory/action research*,* discourse analysis*,* feminist*,* community-based research*,* historical*,* exploratory-descriptive, etc.

4. Methods:

- (observation (non/participant), interview, focus group, case study, life history, narrative, visual ethnographic, theme identification, comparative analysis, cognitive mapping, interpretive methods, document analysis, content analysis, conversation analysis, etc.)

Tracy (2020), *Qualitative Research Methods: Collecting evidence, crafting analysis, communicating impact:*

1. Paradigms (ontology & epistemology assumptions underlie)
   a. Positivist & post-positivist
   b. Interpretive (epistemology: social construction, constructivist, constructionist)
   c. Critical (Ideological; e.g., feminist)
   d. Post-modern (poststructuralism, postmodernism, posthumanism, postcolonialism, postqualitative, critical indigenous inquiry)
2. Theories

- E.g. Symbolic interactionism, Structuration theory, Sensemaking

3. Methodologies
   a. Qualitative (emic/etic, inductive/deductive/abductive; self-reflexivity, context, thick description
   b. Quantitative (etic, deductive)
4. Methods/Approaches:

- Experimental, Case study, Grounded theory, Ethnography, Phenomenology, Participatory action research, Narrative inquiry, Autoethnography, Creative/performative/arts-based

Ponterotto (2005), *Qualitative research in counseling psychology: A primer on research paradigms and philosophy of science:*

1. Methods: Quantitative and Qualitative
2. Paradigms (ontological and epistemological assumptions underlie)
   a. Positivism (Objectivism (epistemology), realism (ontology), deductive, theory verification)
   b. Postpositivism (Critical realism, theory falsification)
   c. Constructivism-Interpretivism (Relativist ontology, subjectivist epistemology)
   d. Critical-Ideological
3. Methodology
   a. Experimental, quasi-experiments, prediction, control
   b. Naturalistic
