# Supplementary material for: Juggling between caregiving and self-actualization: Older parents’ lifelong experience of caring for an adult child with developmental disabilities
Source: PLoS One. 2022 Nov 3;17(11):e0276779. doi: 10.1371/journal.pone.0276779 (PMC9632868; doi:10.1371/journal.pone.0276779)
Supplement: Attachment — Submitted filename: Response to Reviewers.docx [file pone.0276779.s002.docx]

A) Please clarify the sources of funding (financial or material support) for your study. List the grants or organizations that supported your study, including funding received from your institution.

The study was supported by the Shalem Fund for the Development of Services for People with Intellectual Disabilities, grant number: 123/2017

b)        State what role the funders took in the study. If the funders had no role in your study, please state: “The funders had no role in study design, data collection and analysis, decision to publish, or preparation of the manuscript.”

The funders had no role in study design, data collection and analysis, decision to publish, or preparation of the manuscript.

c)        If any authors received a salary from any of your funders, please state which authors and which funders.
The authors did not receive any salary from the founder

d)        If you did not receive any funding for this study, please state: “The authors received no specific funding for this work.”
NA

2. **Please ensure that you refer to Table 1 and 2 in your text**; if accepted, production will need this reference to link the reader to the Table. Done

3. Hila Avieli - https://orcid.org/ 0000-0002-0341-6058

Tova Band-Winterstin - https://orcid.org/ 0000-0002-6381-3661

\Alon Zamir- https://orcid.org/ 0000-0001-6862-3528

**Editor**

| Action | **Editor comment** |
| --- | --- |
| **Done.** | Please ensure that your manuscript meets PLOS ONE's style requirements, including those for file naming. The PLOS ONE style templates can be found at |
| **Done** (pp. 9-10, 18-19). | Please include your tables as part of your main manuscript and remove the individual files. Please note that supplementary tables (should remain/ be uploaded) as separate "supporting information" files. |
| Data availability statement:  The data of this qualitative study contains the narrative of sensitive research subjects (older adults, parents of adults with disabilities), who shared personal and identifying experiences. Participants did not give their consent for datasets to be stored or accessed outside of the research team. Therefore, Haifa University's ethical review board determined that the data set would only be available to the researchers and would not be available to the public. Any queries regarding gaining access to excerpts of the transcripts should be directed to Haifa University IRB at resauth@univ.haifa.ac.il | In your Data Availability statement, you have not specified where the minimal data set underlying the results described in your manuscript can be found. PLOS defines a study's minimal data set as the underlying data used to reach the conclusions drawn in the manuscript and any additional data required to replicate the reported study findings in their entirety. All PLOS journals require that the minimal data set be made fully available. For more information about our data policy, please see http://journals.plos.org/plosone/s/data-availability.  Upon re-submitting your revised manuscript, please upload your study’s minimal underlying data set as either Supporting Information files or to a stable, public repository and include the relevant URLs, DOIs, or accession numbers within your revised cover letter. For a list of acceptable repositories, please see http://journals.plos.org/plosone/s/data-availability#loc-recommended-repositories. Any potentially identifying patient information must be fully anonymized.  Important: If there are ethical or legal restrictions to sharing your data publicly, please explain these restrictions in detail. Please see our guidelines for more information on what we consider unacceptable restrictions to publicly sharing data: http://journals.plos.org/plosone/s/data-availability#loc-unacceptable-data-access-restrictions. Note that it is not acceptable for the authors to be the sole named individuals responsible for ensuring data access.  We will update your Data Availability statement to reflect the information you provide in your cover letter. |
| Orcid ID has been added. | PLOS requires an ORCID iD for the corresponding author in Editorial Manager on papers submitted after December 6th, 2016. Please ensure that you have an ORCID iD and that it is validated in Editorial Manager. To do this, go to ‘Update my Information’ (in the upper left-hand corner of the main menu), and click on the Fetch/Validate link next to the ORCID field. This will take you to the ORCID site and allow you to create a new iD or authenticate a pre-existing iD in Editorial Manager. Please see the following video for instructions on linking an ORCID iD to your Editorial Manager account: https://www.youtube.com/watch?v=_xcclfuvtxQ |
| **Done.** | Please review your reference list to ensure that it is complete and correct. If you have cited papers that have been retracted, please include the rationale for doing so in the manuscript text, or remove these references and replace them with relevant current references. Any changes to the reference list should be mentioned in the rebuttal letter that accompanies your revised manuscript. If you need to cite a retracted article, indicate the article’s retracted status in the References list and also include a citation and full reference for the retraction notice. |
| Data availability statement:  The data of this qualitative study contains the narrative of sensitive research subjects (older adults, parents of adults with disabilities), who shared personal and identifying experiences. Participants did not give their consent for datasets to be stored or accessed outside of the research team. Therefore, Haifa University's ethical review board determined that the data set would only be available to the researchers and would not be available to the public. Any queries regarding gaining access to excerpts of the transcripts should be directed to Haifa University IRB at [resauth@univ.haifa.ac.il](mailto:resauth@univ.haifa.ac.il) | The PLOS Data policy requires authors to make all data underlying the findings described in their manuscript fully available without restriction, with rare exception (please refer to the Data Availability Statement in the manuscript PDF file). The data should be provided as part of the manuscript or its supporting information, or deposited to a public repository. For example, in addition to summary statistics, the data points behind means, medians and variance measures should be available. If there are restrictions on publicly sharing data—e.g. participant privacy or use of data from a third party—those must be specified. |

**Reviewer 1:**

| **Action** | **Reviewer comment** |
| --- | --- |
| **Done,** the word was changed throughout the article. | Thank you for doing this research. It is an interesting span of experts that is being used for this. The use of the word "form" throughout the paper can mean different things in English so it took a few reads to understand the meaning |
| -The definition of self-actualization has been moved to page 2.  - This manuscript adheres to the Journal Article Reporting Standards for Qualitative Research (JARS-Qual; Levitt et al. 2018), which outlines key standards for qualitative writing. The JARS-Qual requires researchers to state their education and experience as do most qualitative writing traditions. | I would have liked more upfront definitions of self actualization over the self promotion of the researchers in how they have done other studies**.** |

**Reviewer 2::**

| **Done (pp. 2).** | “This study aims to explore how parents retrospectively experience and interpret” should say “retrospectively report on and interpret their experience.” | Abstract | |
| --- | --- | --- | --- |
| **Done (pp 1,2):** Juggling between Caregiving and Self-actualization: Older Parents’ Lifelong Experience of Caring for an Adult Child with Developmental Disabilities | Clarify that these are older adult parents of adult children. | Title | |
| **Done (pp. 5-6):** Committed to the examination of how people make sense of major life experiences (Smith et al., 2009, p.1), IPA adopts an interpretive ontological stance. That is, it attempts to uncover the meaning and, in turn, the reality of people’s experiences in the social world. IPA does not view reality as objective, nor is it a methodological approach at the positivist end of the ontological continuum. It does not attempt to define or indeed obtain facts or seek the truth. Rather, it seeks to understand the person’s own experience, the meaning they make of it and, crucially, the interpretation which the researcher makes of the person’s meaning. Smith et al. (2009) describe  IPA as ‘an approach to qualitative, experiential and psychological research  which has been informed by concepts and debates from three key areas of  philosophy of knowledge: phenomenology, hermeneutics and idiography’  (p.11). IPA draws on each of these theoretical approaches to inform its  distinctive epistemological framework and research methodology. | Method” is identified as IPA. Please describe and define IPA in two or three sentences.  Missing is overall research approach. What are the epistemological and ontological assumptions (paradigm), theoretical perspectives, and methodology (research tradition)? The methods flow from this along with the research question. See attached summary. | Method | |
| The required information was added to the manuscripet, (**p. 7):**  Potential participants consented to having their contact information given to the researchers after an introductory telephone call from their case manager at the NGO. Only parents who gave their initial consent were approached by the researchers.  Table 1  Although participants agreed to the specific information in the table being published, we do agree with the Reviewer’s comment that it may be too identifying, due to the small sample size. Thus, Table 1’s content was changed to include less revealing personal information. We now believe that the participants’ anonymity is protected. | “receiving a list of potential participants” from NGO. Did potential participants (service users?) consent to having their name/contact information given to researchers?  Table 1: Did the participants confirm that including those details would not identify them or they were agreeable to those details included? Is the population large enough to disguise individuals with these characteristics? | Ethics |  |
| *Member checking*, also known as participant or respondent validation, is a technique for exploring the credibility of results. Data or results are returned to participants to check for accuracy and resonance with their experiences. In the currents study, we had a second session with five of the participants in which we presented the findings and received feedback.  Birt, L., Scott, S., Cavers, D., Campbell, C., & Walter, F. (2016). Member checking: a tool to enhance trustworthiness or merely a nod to validation? Qualitative Health Research, 26(13), 1802-1811. <https://doi.org/10.1177/1049732316654870> | Good description. Transparent. Inductive and deductive acknowledged.  What does member checking mean and how was it operationalized? | Analysis |  |
| -We have now added a Theme Summary  table at the end of the Findings section as  requested, including the number of  participants represented in each theme. In accordance with Reviewer 1’s comment regarding protecting the participants’ anonymity, we have refrained from identifying which pattern corresponds with any specific participant.  - Other issues that were mentioned in the  interviews and were not relevant to  parental caregiving are beyond the scope of the current manuscript, and thus were not included.  - Data analysis and was performed  according to Interpretive Phenomenological  Analysis (IPA) method, which stipulates that while the analysis may include the entire data set, the Findings section will include only those quotes  which best illustrate the phenomenon at  hand:  Researchers should seek only  quotations that illustrate the findings…  Furthermore, to put flesh on the bones, the quotations may represent a selection,  accurately reproduced, rather than the  entire dataset.  Eldh, A. C., Årestedt, L., &amp; Berterö, C. (2020).  Quotations in Qualitative Studies:  Reflections on Constituents, Custom, and  Purpose. International Journal of Qualitative  Methods.  <https://doi.org/10.1177/160940692096926> | Please include summary at the beginning or end of this section with one to two sentence describing and comparing the four themes.  Include a figure of the four themes and their relationships/comparisons/contrasts.  Please include one quote from each of 15 participants.  Please clarify and put in Table 1 which parents fit which category and if they fit more than one. Was there any other pattern noted?  Further clarify differences between total devotion and self-actualizaton through caregiving. | Findings |  |
| 1. **Done** (pp. 18-20). We expanded the dialogue with the literature. Further articles have been added.   pp. 18 -The critical approach to disability studies emphasizes the importance of self-actualization in the lives of people with disabilities and their families. This approach argues that limitation should not be an obstacle to self-actualization. In the past, the attitude of the disabled person and his family was based on the “Tragedy model, which perceived life with a disability as a life of suffering and dissatisfaction (Goldiner, 2022). This study, following various studies in recent years, focused on the positive aspects alongside the difficulties of living with a person withdisability.  The analysis of the findings reveal four patterns of parental experiences regarding the issue of self-actualization alongside a CWDD.  pp 20**:** In this context, it is worth quoting the well-known psychologist Erich Fromm (2000), who claims in his book "The Art of Love" that the ability to love another person constitutes self-realization and that love is created through care and devotion.   2. **Done (**pp. 22**):** The research findings indicate there is no unified profile of parental perceptions of self-actualization while living and aging with a CWDD. The different narratives presented in this study can serve as a framework for interventions that will meet the parents’ needs at this stage of their life. Professionals (such as social workers, and psychotherapists) should pay special attention to parents who display signs of burden of care, and encourage them to try to develop and fulfill self-actualization needs, in order to facilitate closure processes. Life review processes [9] may be bridled to strengthen the existing understanding of the choices that were made.   In addition, we recommend creating a support group for aging parents of a person with CWDD. This type of group can provide parents with the support of other parents who are in the same situation. We believe these groups could serve as a platform for the critical issue of self-realization/actualization(?) alongside limitation.  In conclusion, this article emphasizes the importance of a sense of self-actualization among parents providing care to a person with DD Despite the great importance of this topic, there is very little research on it. We call for greater emphasis to be placed on designing intervention programs that take this issue into account.   3. **Done.** pp. 22:   In conclusion, this article emphasizes the importance of a sense of self-actualization among parents providing care to a person with DD. Despite the great importance of this topic, there is very little research on it. We call for greater emphasis to be placed on designing intervention programs that take this issue into account. | 1.Further dialogue with the literature is needed  .  2.Expand on the implications and relate to current interventions. Which professions would they impact? Include a conclusion;  3.article ends abruptly. | Discussion |  |
| **Done.** | Minor spelling, grammar, punctuation for final review after revisions. | Writing |  |
| Thanks for referring to the articles. The following articles have been added to the Literature Review (pp. 4-7). | Consider removing Google Books links.  Some authors are missing.  Consider additional references on positive parental experiences:  Beighton, C., & Wills, J. (2017). Are parents identifying positive aspects to parenting their child with an intellectual disability or are they just coping? A qualitative exploration. Journal of Intellectual Disabilities, 21(4), 325–345. https://doi.org/10.1177/1744629516656073  Green, S. E. (2007). “We’re tired, not sad”: Benefits and burdens of mothering a child with a disability. Social Science and  Medicine, 64(1), 150–163. https://doi.org/10.1016/j.socscimed.2006.08.02  Matthews, E. J., Puplampu, V., & Gelech, J. (2021). Tactics and strategies of family adaptation among parents caring for children and youth with developmental disabilities. Global Qualitative Nursing Research: https://journals.sagepub.com/doi/pdf/10.1177/23333936211028184  Schall, C. (2000). Family perspectives on raising a child with Autism. Journal of Child and Family Studies, 9(4), 409–423. https://doi.org/10.1023/A%3A1009456825063 | References |  |
