# Supplementary material for: Juggling between caregiving and self-actualization: Older parents’ lifelong experience of caring for an adult child with developmental disabilities
Source: PLoS One. 2022 Nov 3;17(11):e0276779. doi: 10.1371/journal.pone.0276779 (PMC9632868; doi:10.1371/journal.pone.0276779)
Supplement: Attachment — Submitted filename: 082222review.docx [file pone.0276779.s003.docx]

Ethics Statement: Please write out what NGO stands for prior to only using its acronym. Readers outside of Israel may not know what this is.

Page 4: “his needs” should be changed to gender neutral phrasing.

Page 5: “his professional or personal goals” should be changed to gender neutral phrasing. The term his is used throughout and should be adjusted.

Page 6: run on sentence: Years of intense caregiving shapes and colors parents’ identity throughout the life course [51]; self-actualization may serve as the goal of life review, by creating closure and achieving self-acceptance for these parents [29].

Page 7: Please write out what NGO stands for at the first introduction of the acronym.

Table 1: were participants personal identifying information disclosed? I see this somewhat addressed in ethical considerations, however did you assign random names to participants?. How has the data collection and discussion remained anonymous to protect this population?

Page 18: Formatting on Discussion.

Page 24: Formatting.
