# Supplementary material for: Juggling between caregiving and self-actualization: Older parents’ lifelong experience of caring for an adult child with developmental disabilities
Source: PLoS One. 2022 Nov 3;17(11):e0276779. doi: 10.1371/journal.pone.0276779 (PMC9632868; doi:10.1371/journal.pone.0276779)
Supplement: Attachment — Submitted filename: Response to Reviewers 11.10,22.docx [file pone.0276779.s004.docx]

**Response to Reviewers**

**Editor**

| Action | **Editor comment** |
| --- | --- |
| The list of refrences was carefully checked.  We removed a single article that was incorrectly cited and therefore could not be located (Page 7) and replaced it with the following one:  Brown S, Brown R, Preston S. A model of human caregiving motivation. Moving beyond self-interest: Perspectives from evolutionary biology, neuroscience, and the social sciences 2011:75-88. https://doi.org/10.1080/00332747.1963.11023339 | Please review your reference list to ensure that it is complete and correct. If you have cited papers that have been retracted, please include the rationale for doing so in the manuscript text, or remove these references and replace them with relevant current references. Any changes to the reference list should be mentioned in the rebuttal letter that accompanies your revised manuscript. If you need to cite a retracted article, indicate the article’s retracted status in the References list and also include a citation and full reference for the retraction notice. |

**Reviewer 1:**

| **Action** | **Reviewer comment** |
| --- | --- |
| **Done**.  **Page 7.** | Please write out what NGO stands for prior to only using its acronym. Readers outside of Israel may not know what this is. |
| **Done.** Thank you very much for the careful reading, we have now corrected gender bias language throughout. | Page 4: “his needs” should be changed to gender neutral phrasing.  Page 5: “his professional or personal goals” should be changed to gender neutral phrasing. The term his is used throughout and should be adjusted. |
| **Done.**  **Page 6.**  Contemplating self-actualization is a significant issue in the life review process, in which parents reflect and give retrospective meaning to past experiences and choices, in light of their current life's consequences [10], [40]. Years of intense caregiving shapes and colors parents’ identity throughout the life course [51]. Life review, includes looking at the past and examining their choices [29]. Thus, parents of older adults with disabilities may reflect on choices that were made which affected their caregiving to the CWDD, as well their self-actualization and the interplay between these two concepts. | Page 6: run on sentence: Years of intense caregiving shapes and colors parents’ identity throughout the life course [51]; self-actualization may serve as the goal of life review, by creating closure and achieving self-acceptance for these parents [29]. |
| **Done**. | Page 7: Please write out what NGO stands for at the first introduction of the acronym. |
| Table 1: In order to protect the anonymity of the participants pseudo names were used to describe the participants and their families. Also, in some cases the gender of the participant's family member was changed to further anonimyze the data.  A comment regarding the used of pseudo names and gender change was inserted in page 8 and in table 1.  Moreover, in line with the reviewers' comment, we have choose to present some very general data regarding the families (e.g. we did not include occupations and residenchial aria). We believe that using pseudo names, using gender change, and not including some identifying details will provide our participants with anonymity. | Table 1: were participants personal identifying information disclosed?   1. I see this somewhat addressed in ethical considerations, however did you assign random names to participants? 2. . How has the data collection and discussion remained anonymous to protect this population? |
| **Done.** | Page 18: Formatting on Discussion.  Page 24: Formatting. |
